# Fluid Overload in Pediatric Univentricular Patients Undergoing Fontan Completion

**DOI:** 10.3390/jcdd10040156

**Published:** 2023-04-05

**Authors:** Victorien A. C. Luppes, Ariane Willems, Mark G. Hazekamp, Nico A. Blom, Arend D. J. Ten Harkel

**Affiliations:** 1 Department of Pediatric Cardiology, Leiden University Medical Center, 2333 ZA Leiden, The Netherlands; v.a.c.luppes@lumc.nl (V.A.C.L.);; 2 Pediatric Intensive Care Unit, Department of Intensive Care, Leiden University Medical Center, 2333 ZA Leiden, The Netherlands; 3 Department of Cardiothoracic Surgery, Leiden University Medical Center, 2333 ZA Leiden, The Netherlands

**Keywords:** congenital heart disease, Fontan, univentricular, cardiac surgery, TCPC, pediatric intensive care unit, fluid overload, length of stay, mechanical ventilation, cardiac events

## Abstract

Background: Fluid overload (FO) is known to occur frequently after pediatric cardiac surgery and is associated with morbidity and mortality. Fontan patients are at risk to develop FO due to their critical fluid balance. Furthermore, they need an adequate preload in order to maintain adequate cardiac output. This study aimed to identify FO in patients undergoing Fontan completion and the impact of FO on pediatric intensive care unit (PICU) length of stay (LOS) and cardiac events, defined as death, cardiac re-surgery or PICU re-hospitalization during follow-up. Methods: In this retrospective single center study, the presence of FO was assessed in 43 consecutive children undergoing Fontan completion. Results: Patients with more than 5% maximum FO had an extended PICU LOS (3.9 [2.9–6.9] vs. 1.9 [1.0–2.6] days; *p* < 0.001) and an increased length of mechanical ventilation (21 [9–121] vs. 6 [5–10] h; *p* = 0.001). Regression analysis demonstrated that an increase of 1% maximum FO was associated with a prolonged PICU LOS of 13% (95% CI 1.042–1.227; *p* = 0.004). Furthermore, patients with FO were at higher risk to develop cardiac events. Conclusions: FO is associated with short-term and long-term complications. Further studies are needed to determine the impact of FO on the outcome in this specific population.

## 1. Introduction

Fluid overload (FO) is known to occur frequently after pediatric cardiac surgery and is due to several factors including pre-existent heart failure, postoperative inflammation, capillary leakage and decreased renal function [1]. It can be described as a state of edema, hypervolemia and excessive weight gain in patients who receive fluid therapy. FO is associated with several outcomes, including prolonged mechanical ventilation (PMV) and pediatric intensive care unit (PICU) length of stay (LOS), low cardiac output syndrome (LCOS) and increased mortality, in pediatric patients after cardiac surgery [2,3,4,5,6]. It is also associated with acute kidney injury (AKI) and renal replacement therapy (RRT) [7,8].

Patients undergoing Fontan surgery are potentially at increased risk of developing FO due to their strong dependence on adequate preload for proper hemodynamics. Nowadays, functional single ventricle patients are mostly palliated with a total cavo-pulmonary connection (TCPC). They either receive an extracardiac conduit (ECC) or an intra-atrial lateral tunnel (ILT). Early mortality and morbidity after the Fontan operation have decreased considerably due to improved surgical techniques and postoperative care management [9]. However, with time, these patients can develop several cardiac morbidities. A Fontan circulation in particular with passive flow through the pulmonary arteries has an increased risk of venous congestion and limited flow resulting in a critical fluid balance [10]. Adequate preload after surgery is crucial to maintain cardiac output while patients are at risk of venous congestion, oedema and ascites. This critical fluid balance results in a vulnerability to develop FO after Fontan completion.

To our knowledge, there has been no study performed that investigated FO in patients undergoing Fontan surgery. The aim of this research was to study the presence of postoperative FO in pediatric patients undergoing TCPC surgery and its effect on PICU LOS as well as cardiac events during follow-up. Our secondary aim was to investigate which preoperative factors were associated with the risk of developing FO.

## 2. Materials and Methods

A retrospective cohort study of all pediatric univentricular patients who underwent a TCPC and were admitted to the PICU from January 2014 until January 2022 in the Leiden University Medical Center (LUMC) was performed. The cohort comprised 43 consecutive patients. Provided surgical care and intensive care management were equivalent during the study period.

The total daily fluid balance was obtained from our electronical patient record that automatically extracts the total daily output (urine, losses via gastro-intestinal tract, blood losses and drain losses including thoracic drains and abdominal drains) from the total daily input (administered fluids, blood products, parenteral nutrition and oral intake). Insensible fluid loss from the skin and respiratory system was not included in the daily fluid balance. Fluid intake, fluid output, fluid balance, urine output and drain losses were obtained cumulatively per day, starting at the day of surgery (DOS) up to postoperative day (POD) 7, resulting in a postoperative study period of 7 days. FO was calculated for each patient per day using the following method: [(total fluid in (L)–total fluid out (L))/preoperative weight (kg)] × 100%. The maximum percentage of daily cumulative FO per patient in the whole postoperative study period including DOS was expressed as maximum fluid overload (maxFO). We used a cutoff point of 5% maxFO for our analyses, consistent with previous literature on pediatric cardiac populations [3,7]. Subsequently, the cumulative fluid overload (cFO) on POD 1 and POD 2 was calculated. This was computed by accumulating the daily cumulative FO of DOS and POD 1, and by accumulating the daily cumulative FO of DOS, POD 1, and POD 2, respectively. As FO often occurs shortly after surgery and most patients were discharged after POD 2, cFO was not calculated for POD 3 and thereafter.

Besides baseline demographics, various preoperative, intra-operative and postoperative variables were collected. Preoperative data included demographic data and hemoglobin and serum creatinine levels. Patients with hypoplastic left heart syndrome as diagnosis and those who required either a pulmonary artery banding (PAB) or a systemic-pulmonary shunt prior Glenn were noticed. Preoperative cardiac catheterization and cardiac ultrasound findings for screening prior to Fontan completion obtained after the Glenn shunt were collected. Mean pulmonary artery pressure (mPAP), presence of significant collaterals and interventions such as coiling of significant collaterals and dilatations of narrowed vessels during cardiac catheterizations were obtained. Collaterals were defined as significant when they were closed during cardiac catheterization. Cardiac function and valvular insufficiencies and stenosis were extracted from cardiac ultrasound reports. Intra-operative data included cardiopulmonary bypass (CPB) time, aortic cross-clamp (AOX) time, minimum body temperature, amount of ultrafiltration (UF) and total administered erythrocytes and fresh frozen plasma (FFP/Omniplasma). Postoperative PICU variables included PICU LOS, length of invasive ventilation, lowest and highest hemoglobin during PICU stay, maximum lactate, lowest serum albumin and highest serum creatinine. Invasive ventilation of more than 48 h was defined as prolonged invasive ventilation. Duration and the cumulative production of thoracic and abdominal drains during PICU stay were collected. The maximum vasoactive–inotropic score (VIS) was calculated for each patient during PICU stay as follows: maximum dobutamine + (100 × maximum epinephrine) + (10 × maximum milrinone) + (100 × maximum norepinephrine) [10]. At our center, neither dopamine nor vasopressin were used for inotropic support in the postoperative period. All dosages were in mcg/kg/min. Furthermore, acute kidney injury (AKI) was defined by means of criteria from the Acute Kidney Injury Network and Kidney Disease Improving Global Outcomes group [11]. We used the criteria for serum creatinine to calculate the different AKIN stages that corresponds to AKI. We used preoperative serum creatinine as baseline serum creatinine, often derived the day before surgery. Besides this, maximum serum creatinine levels during the entire postoperative PICU stay were used to calculate the AKIN stages. The usage of this scoring system has been validated in infants who underwent congenital heart surgery [12].

Regarding follow-up, development of cardiac events was obtained from electronical patient records. Cardiac events were defined as at least one of the following complications: mortality, TCPC takedown, cardiac re-surgery, or cardiac-related PICU re-hospitalization. These cardiac events were analyzed as a composite outcome variable and included if present between TCPC surgery until May 2022, the end of the follow-up period. Cardiac re-surgery was defined as any major cardiac-related surgery after discharge of the TCPC-related PICU hospitalization. Minor cardiac or thoracic surgeries such as placement of thoracic drains or delayed closure of the sternum were excluded from present analysis. Cardiac-related PICU re-hospitalization was defined as any cardiac-related PICU re-hospitalization after discharge of the TCPC-related PICU hospitalization. In patients presenting multiple cardiac events during follow-up, only the first cardiac event after TCPC surgery was considered in the analysis.

Normally distributed data are presented as means (standard deviations) and non-normally distributed data are presented as medians [interquartile range]. Categorical data are presented as its frequency and percentage. Normal probability plots and Shapiro–Wilk tests were used to determine whether data are normally distributed or not. Data were compared between two groups based on percentage of maximum FO using *t*-tests for normally distributed continuous variables and Mann–Whitney U tests for non-normally distributed continuous variables. Fisher’s exact tests were used to analyze categorical variables.

To examine the independent relation between maximum FO and PICU LOS, we performed a multivariate linear regression analysis. As PICU LOS was skewed, we used log10 transformation to obtain normally distributed data to perform linear regression analyses. Variables that were significantly associated to PICU LOS in the univariate linear regression analyses (*p*-value threshold of <0.1) were added to the multiple linear regression analysis using a mixed selection approach. Besides maximum FO, we added AoX time, total administered erythrocytes during surgery, maximum lactate during PICU admission, lowest hemoglobin, lowest albumin, maximum creatinine, the usage of an abdominal catheter and the VIS score to the multivariate model. After the mixed selection approach, maximum lactate and maximum creatinine were obtained from the multivariate regression as covariate adjustments to determine the independent relation of the maximum FO to PICU LOS. The results of both univariate and multivariate linear regression analyses were transformed using the base 10. Coefficients accompanied by their 95% confidence intervals were outlined. Kaplan–Meier log-rank pooled comparison was used to analyze the development of cardiac events.

IBM SPSS Statistics version 25 release 25.0.0.2 was used to perform all analyses. Statistical significance was defined as a *p*-value less than 0.05.

## 3. Results

In total, 43 consecutive patients underwent a TCPC and were included in the study. All but one patient underwent a TCPC with a fenestration between the extracardiac conduit and the atrium. Of the 43 patients, 9 (21%) patients had hypoplastic left heart syndrome (HLHS). One patient was born prematurely, and one patient had a gene defect known as a variance in the NOTCH-1 gene. At the time of surgery, patients had a median weight of 15.6 kg, a median length of 102 cm and a median age of 4.1 years. Most patients (65%) were male. Shortly before surgery, patients had a median serum creatinine of 31 µmol/L and a mean hemoglobin of 10.3 mmol/L. Whitin the cohort of 43 patients, we defined 2 groups based on maximum FO, 23 (53%) patients had a maxFO of less than 5% and 20 (47%) patients had a maxFO of more than 5%. Demographics and preoperative variables of both groups are presented in Table 1 and were not significantly different between both groups.

### 3.1. Intra-Operative Variables

Median CPB time was 125 min and median AOX time was 74 min. Patients in the more than 5% maxFO group had a significant longer median AOX time compared to the lower than 5% maxFO group, respectively, 79 min vs. 53 min, while CPB time was not significantly prolonged in this group. Furthermore, patients in the more than 5% maxFO group received more UF (Table 1).

### 3.2. Postoperative PICU Variables

Mean maxFO was 5.3 (2.9)% in the entire study population. As shown in Table 1, patients with more than 5% maxFO had a prolonged PICU LOS and duration of invasive ventilation. About 45% of the patients in the more than 5% maxFO group required prolonged invasive ventilation compared to 4% in the group of patients that had less than 5% maxFO. Drain duration as well as total drain production was significantly expanded in the group of patients with more than 5% maxFO. The group with high maxFO required more intake fluids compared to the low maxFO group. Patients with more than 5% maxFO had also significantly higher hemoglobin values during PICU stay, higher lactate, higher VIS, lower serum albumin values and were more likely to have an abdominal tube or drain. As set out in Table 1, 11 patients (26%) met the criteria for AKI according to the AKIN criteria. Patients in the more than 5% maxFO group had a significantly higher peak serum creatinine and nearly 40% met the criteria for AKI. However, none of the patients required RRT, including peritoneal dialysis.

### 3.3. Regression Analysis

To further evaluate the association between FO and a prolonged PICU LOS, a linear regression analysis was performed. Univariate linear regression analysis resulted in predictors for PICU LOS and are outlined in Table 2. The analysis demonstrated that a longer AOX time, a higher number of administered erythrocytes during surgery, a higher creatinine and lactate levels during PICU stay, the requirement of an abdominal tube or drain during PICU stay, and higher VIS scores during PICU stay were associated with an extended PICU LOS. Furthermore, various parameters of FO were associated with a longer PICU LOS of which maxFO had the strongest association. After multivariate linear regression, which is outlined in Table 3, maximum lactate during PICU stay was obtained as covariate adjustment to determine the independent relation of the maxFO to PICU LOS. For each increase of 1% of maxFO, PICU LOS was prolonged by 13%.

### 3.4. Follow-Up

**Figure 1:**
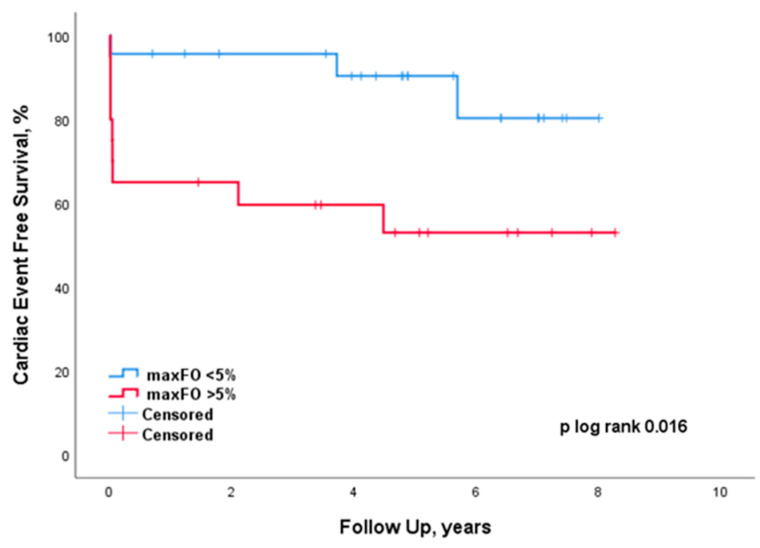
Development of cardiac events according to maximum percentage of FO.

Development of cardiac events is presented in Figure 1. Median follow-up time was 5.1 years [3.5–7.0]. Nine (45%) patients developed cardiac events in the more than 5% maxFO group in comparison to only three (13%) patients in the less than 5% maxFO group (*p*-value of 0.016). PICU re-hospitalization after TCPC-related PICU dismissal included complications derived from arrhythmias. One patient had atrial fibrillation and one patient had a small complex tachycardia. One of these patients had cardiac reoperation for new pacemaker implantation after PICU re-hospitalization, of which only the PICU re-hospitalization was included in the analysis. Eight patients had cardiac re-surgery after TCPC-related PICU dismissal and this included substitution of the extracardiac conduit, myectomy and tricuspid valve repair, new pacemaker placement, drainage of cardiac tamponade, pulmonary thrombectomy, drainage of pleural effusion, ECMO and resolution of sub-aortal obstruction. Most of these patients (7) were submitted to the PICU after surgery; however, these PICU re-admissions are not included in the analysis as they already entered the composite outcome regarding their cardiac reoperation. There were two in-hospital deaths after TCPC surgery, of which one patient was already included in the analysis due to cardiac reoperation. Furthermore, there was one TCPC takedown during TCPC-related PICU stay. When we evaluated only the short-term outcome before hospital discharge, maxFO was a significant risk factor to develop cardiac events as well (*p* = 0.010).

### 3.5. Preoperative Cardiac Catheterizations, Ultrasounds and Interventions

Mean pulmonary artery pressure (mPAP) (10.3 (3.9) vs. 10.6 (3.7); *p* = 0.779), the presence of significant collaterals (8 vs. 11; *p* = 0.756), interventions such as coiling of significant collaterals (8 vs. 9; *p* = 1.000) and dilatations of narrowed vessels (1 vs. 2; *p* = 1.000) during cardiac catheterizations were not significantly different between the group of patients with more than 5% maxFO vs. the group of patients with less than 5% maxFO. Mean mPAP for all patients was 10.5 (3.7) mmHg. Furthermore, all patients had a good cardiac function as established by the ultrasound prior to surgery. The group of patients with more than 5% maxFO did not show significantly more valvular insufficiencies (4 vs. 2; *p* = 0.390) or stenosis (1 vs. 2; *p* = 1.000). The number of interventions of PAB (9 in maxFO group vs. 11; *p* = 1.000) and systemic-pulmonary shunts prior to the Glenn shunt (13 in maxFO group vs. 18; *p* = 0.504) did not significantly differ in both groups.

## 4. Discussion

Our study found that in children undergoing Fontan surgery, a postoperative maxFO of more than 5% was associated with prolonged invasive mechanical ventilation, prolonged PICU LOS and increased cardiac events during follow-up including death, TCPC takedown, cardiac surgery and PICU re-hospitalization. Our results also showed that patients with a maxFO of more than 5% had significant longer AoX times, higher creatinine levels postoperative and higher VIS. These associations do not directly indicate that FO has a cause–effect relationship to the reported outcomes. Fontan patients with a higher postoperative FO were probably in need of larger amounts of fluid to maintain adequate blood pressure and cardiac output. In this context, in a highly preload dependent population, FO which is usually seen as a negative parameter could be referred to as a prerequisite for these patients. To determine whether FO is associated with preoperative factors, we assessed multiple factors that may be related to postoperative hemodynamic instability. We found, however, no significant difference in cardiac condition derived from cardiac catheterization, cardiac ultrasound and interventions prior to Fontan surgery. Another complicating factor is that FO is closely related to other clinical parameters such as VIS score and renal function. Nonetheless, a multivariate analysis was performed to adjust for possible confounders and determine that maxFO was independently associated with prolonged PICU LOS. Whether or not FO is a risk marker or a risk factor in this specific population, a prolonged LOS should be taken into account as well as the increased risk of complications.

Critically ill patients are vulnerable to develop FO due to an increased capillary leak, which facilitates administered intravenous fluids to enter the extracellular space and cause edema and fluid overload [13]. Consequently, cerebral edema can result in delirium, myocardial edema in limited contractility and diastolic dysfunction, pulmonary edema in reduced gas exchange, renal interstitial edema in diminished GFR and tissue edema in surgical site infection and delayed healing [13,14]. Pediatric patients are at risk to develop FO after cardiac surgery due to pre-existent heart failure, complex congenital heart defects and decreased renal function [1]. Especially fluid management after Fontan completion is a delicate balance. On one side, FO can result in pulmonary effusion causing ventilation difficulties and myocardial edema causing decreased cardiac output or kidney injury, among others. On the other side, adequate preload is essential in a univentricular circulation to support sufficient cardiac output, adequate renal perfusion and maintain systemic blood pressure [13]. Measures related to FO at the bedside can be meaningful in optimizing postoperative care after Fontan surgery due to its critical fluid balance. Closely monitoring of the fluid balance and early detection of FO is important. After initial fluid resuscitation and ensuring adequate preload, a restrictive fluid strategy, forced diuresis with diuretics and early initiation of fluid removal in AKI patients by RRT should be considered. Peritoneal Dialysis (PD) is sometimes used in pediatric cardiac surgery patients to improve hemodynamic instability. PD was shown to be a safe and effective measure for pediatric patients after cardiac surgery with CPB requiring RRT [15]. Abdominal decompression and abdominal fluid removal in patients with a PD catheter can have positive effects on the degree of FO and therefore the development of adverse events. Furthermore, a restrictive fluid management can be considered as an alternative method to prevent and treat FO. Although it can be effective, conservative fluid management has several disadvantages in univentricular patients. Restrictive fluid therapy can result in difficulties to maintain adequate preload and blood pressure, and consequently lead to the extended use of vaso-active inotropies. There is no consensus about the initiation of restrictive fluid management after both adult and pediatric cardiac surgery because there have been no randomized controlled trials [16]. In critically ill pediatric patients, randomized clinical trials do not exist [17,18,19]. The above-discussed measures in preventing FO need to be validated in well-designed prospective studies.

Previous studies indicated that, in critically ill children requiring RRT, a FO above 10% to 20% was related to adverse outcomes including mortality [20,21,22]. In critically ill pediatric and neonatal septic shock patients, a threshold of 10% FO has been acknowledged as a critical point to act upon by The American College of Critical Care Medicine [23]. There is no consensus about the degree of FO that is related to morbidity and mortality in the cardiac patient population, and specifically not in pediatric patients with congenital heart disease. In addition, studies are difficult to compare due to both different methods to calculate and different ways to report FO, i.e., cumulatively or maximum/peak FO. FO can be calculated using the method described by Goldstein et al. that uses patients’ fluid balance and admission weight [24]. An alternative method to calculate the degree of FO is based on patients’ weight at PICU admission and subsequently daily weight. However, FO calculated by this weight-based method was associated to mortality similarly to FO calculated by the fluid-based method in a broad PICU population [25]. We calculated the degree of FO in our study cohort using the fluid-based method, as our patients were not weighed daily. Our study demonstrated that more than 5% maximum FO was associated with adverse events. This threshold of 5% FO is substantially lower than the literature in critically ill pediatric patients. We might expect a higher overall percentage of FO due to the high RACHS-1 risk score of our patient cohort and their pre-existent critical fluid balance. However, our results are in line with other studies in congenital heart surgery that reported 5% maximum FO and 5% FO cumulatively on POD 1 or POD 2 to be associated with increased morbidity [2,3,7]. Other studies in pediatric cardiac surgery patients reported increased morbidity related to higher FO thresholds of 16% and 12% [4,8]. In order to compare studies more easily, standardized methods to calculate FO and report FO should be embedded.

This study has several strengths. To our knowledge, it is the first study to look at the association of FO and outcome in univentricular patients after Fontan completion. As a novel approach, we aimed at investigating certain long-term follow-up parameters in our study cohort and relate them to the amount of FO that patients have had during their PICU stay after cardiac surgery. In comparison, various previous studies investigated exclusively the peri-operative effects of FO in pediatric patients following congenital heart surgery. However, this study has some limitations. Insensible fluid loss was not included in our daily fluid balance. Furthermore, this study included a small number of patients, although it was a homogeneous population solely undergoing the ECC-TCPC. We, therefore, used a composite outcome of cardiac events similar to the outcome used in the study of Hazle et al. [4] to allow comparability of results between studies and to remain powered enough in our statistical analysis. Due to the retrospective character of the study, there is an increased risk of confounding, bias and missing data. However, in the case of our study, missing data were infrequent, and a multivariate analysis was performed to adjust for possible confounders. Future research should expand on pulmonary function. Fontan patients are highly dependent on sufficient pulmonary compliance due to the lack of a ventricular force. Pulmonary vascular impedance and adequate development of the pulmonary system are important for an efficient and sustainable Fontan circulation [26]. Deterioration in pulmonary function could be damaging and should be taken into consideration in future research.

In conclusion, this study shows that FO is associated with short-term and long-term complications in univentricular patients following TCPC surgery. However, it does not mean causality. FO is probably rather a risk marker than a risk factor due to the highly preload dependent population and strong associated factors to FO. Nevertheless, our study provides insight in the relatively low amount of FO postsurgery in Fontan patients and presents awareness of which patients are at high risk of complications. Further studies are needed to determine adequate fluid management in this population.

## Figures and Tables

**Table 1:** Demographics, preoperative, intra-operative and postoperative parameters by maximum percentage of fluid overload.

Variables	All (N = 43)	maxFO < 5% (N = 23)	maxFO > 5% (N = 20)	*p*-Value
Demographics and Preoperative				
Male, N(%)	28 (65)	15 (65)	13 (65)	1.000
Age, years	4.1 [3.5–4.7]	4.1 [3.5–5.0]	4.0 [3.6–4.6]	0.805
Weight, kg	15.6 [15.0–16.6]	15.7 [15.0–18.9]	15.6 [15.0–16.6]	0.608
Length, cm	102 [98–107]	102 [100–110]	101 [98–105]	0.336
Hemoglobin, mmol/L	10.3 (1.1)	10.3 (1.0)	10.4 (1.2)	0.716
Creatinine, µmol/L	31 [28–39]	29 [27–38]	32 [29–40]	0.369
Prematurity, N(%)	1 (2)	1 (4)	0 (0)	1.000
Chromosomal abnormalities, N(%)	1 (2)	1 (4)	0 (0)	1.000
Hypoplastic left ventricle, N(%)	9 (21)	3 (13)	6 (30)	0.263
Intra-Operative				
CPB, min	126 [105–185]	125 [92–173]	140 [109–206]	0.337
AOX, min	75 [24–100]	53 [0–88]	79 [69–107]	0.019
Minimum temperature, °C	26.4 (4.1)	26.4 (4.9)	26.4 (3.5)	0.990
Ultrafiltration, mL	600 [323–840]	480 [248–800]	710 [500–1120]	0.041
Administered erythrocytes, mL	100 [0–250]	50 [0–213]	150 [0–275]	0.145
Administered FFP, mL	160 [68–255]	170 [15–300]	160 [75–220]	0.995
Postoperative				
PICU LOS, days	2.6 [1.8–4.0]	1.9 [1.0–2.6]	3.9 [2.9–6.9]	<0.001
Invasive ventilation, hours	9 [6–22]	6 [5–10]	21 [9–21]	0.001
Prolonged ventilation > 48 h, N(%)	10 (23)	1 (4)	9 (45)	0.003
Maximum lactate, mmol/L	2.8 [2.4–3.6]	2.5 [2.3–3.4]	3.2 [2.6–3.7]	0.047
Lowest hemoglobin, mmol/L	7.1 (0.9)	7.2 (0.8)	7.0 (1.0)	0.551
Highest hemoglobin, mmol/L	8.7 (1.1)	8.2 (1.0)	9.2 (1.0)	0.003
Lowest serum albumin, g/L	29 (5)	32 (4)	25 (5)	<0.001
Highest creatinine, µmol/L	41 [33–48]	38 [33–43]	44 [37–70]	0.028
Abdominal catheter, N(%)	6 (14)	0 (0)	5 (30)	0.006
AKIN				0.036
Stage 1, N(%)	6 (14)	3 (13)	3 (15)	
Stage 2, N(%)	2 (5)	0 (0)	2 (10)	
Stage 3, N(%)	3 (7)	0 (0)	3 (15)	
VIS score	26 [15–45]	16 [12–31]	44 [23–58]	0.001
Drain duration, days	4 [3–5]	3 [2–4]	4 [4–7]	<0.001
Total drain volume, mL	1027 [550–1886]	610 [400–1027]	1918 [1218–5235]	<0.001
Intake fluids DOS and POD1, mL	2359 [1956–3186]	1976 [1711–2353]	3140 [2604–3803]	<0.001

CPB = cardiopulmonary bypass; AOX = aortic cross-clamp; FFP = fresh frozen plasma; PICU LOS = pediatric intensive care unit length of stay; AKIN = acute kidney injury network; VIS = vasoactive–inotropic score.

**Table 2:** Univariate predictors of PICU LOS.

Predictors	Unstandardized β	95% CI	*p*-Value
AOX	1.007	1.002–1.014	0.015
Administered Erythrocytes	1.002	1.000–1.005	0.017
Highest creatinine	1.023	1.012–1.035	0.000
Maximum lactate	1.358	1.164–1.585	0.000
Lowest hemoglobin	0.721	0.532–0.977	0.036
Lowest serum albumin	0.883	0.853–0.916	0.000
Abdominal catheter	4.487	2.286–8.810	0.000
FO DOS (%)	1.122	1.014–1.239	0.026
FO POD1 (%)	1.114	1.035–1.202	0.006
MaxFO (%)	1.178	1.079–1.282	0.000
cFO POD1 (%)	1.096	1.040–1.156	0.001
cFO POD2 (%)	1.059	1.021–1.099	0.004
VIS score	1.014	1.005–1.021	0.002

AOX = aortic cross-clamp; FO DOS = fluid overload on day of surgery; FO POD1 = fluid overload on postoperative day 1; maxFO = maximum fluid overload; cFO POD1 = cumulative fluid overload up and including postoperative day 1; cFO POD2 = cumulative fluid overload up and including postoperative day 2; VIS = vasoactive–inotropic score.

**Table 3:** Multivariate linear regression model for PICU LOS.

Predictors	Unstandardized β	95% CI	*p*-Value
MaxFO (%)	1.132	1.042–1.227	0.004
Maximum lactate	1.271	1.096–1.472	0.002

## Data Availability

The data presented in this study are available upon reasonable request from the corresponding author.
